# Cell-free DNA chromosome copy number variations predict outcomes in plasma cell myeloma

**DOI:** 10.1038/s41408-023-00904-9

**Published:** 2023-09-05

**Authors:** Wanting Qiang, Lina Jin, Tiancheng Luo, Yanchun Jia, Jing Lu, Jin Liu, Haiyan He, Ziliang Qian, Sridurga Mithraprabhu, Yang Liang, Robert Peter Gale, Xia Tao, Depei Wu, Juan Du

**Affiliations:** 1 Department of Hematology, Myeloma & Lymphoma Center, Second Affiliated Hospital of Naval Medical University, 200003 Shanghai, China; 2 Hongyuan Biotech, Biobay, Suzhou, China; 3 Myeloma Research Group, Australian Centre for Blood Diseases, Alfred Hospital-Monash University, Melbourne, VIC Australia; 4 Malignant Hematology and Stem Cell Transplantation, Alfred Hospital, Melbourne, VIC Australia; 5 Department of Hematologic Oncology, State Key Laboratory of Oncology in South China, Collaborative Innovation Center for Cancer Medicine, Sun Yat-sen University Cancer Center, Guangzhou, China; 6 Centre for Haematology, Department of Immunology and Inflammation, Imperial College of Science, Technology and Medicine, London, UK; 7 Department of Pharmacy, Second Affiliated Hospital of Naval Medical University, 200003 Shanghai, China; 8 National Clinical Research Center for Hematologic Diseases, The First Affiliated Hospital of Soochow University, Suzhou, China

**Keywords:** Myeloma, Cancer genomics, Biostatistics

Dear Editor,

Testing for measurable residual disease (MRD) in persons with plasma cell myeloma (PCM) after therapy correlates with therapy outcomes including progression-free survival (PFS) and survival (reviewed in refs. [1, 2]). Most MRD-testing in this setting uses multi-parameter flow cytometry (MPFC) but prediction accuracy is imperfect with C-statistics of only about 0.67 for PFS and 0.76 for overall (OS) [3]. Recently, MRD-testing includes next-generation sequencing (NGS) and/or next-generation flow cytometry (NGFC) [4, 5]. These approaches are more sensitive compared with MPFC but there are no data on C-statistics for these tests. Prediction inaccuracies arise from diverse sources such as inadequate sensitivity and/or specificity, imprecision and sampling biases resulting from non-homogeneous bone marrow involvement and/or extra-medullary PCM [6, 7]. More accurate methods to predict PFS and OS are needed.

**Fig. 1:**
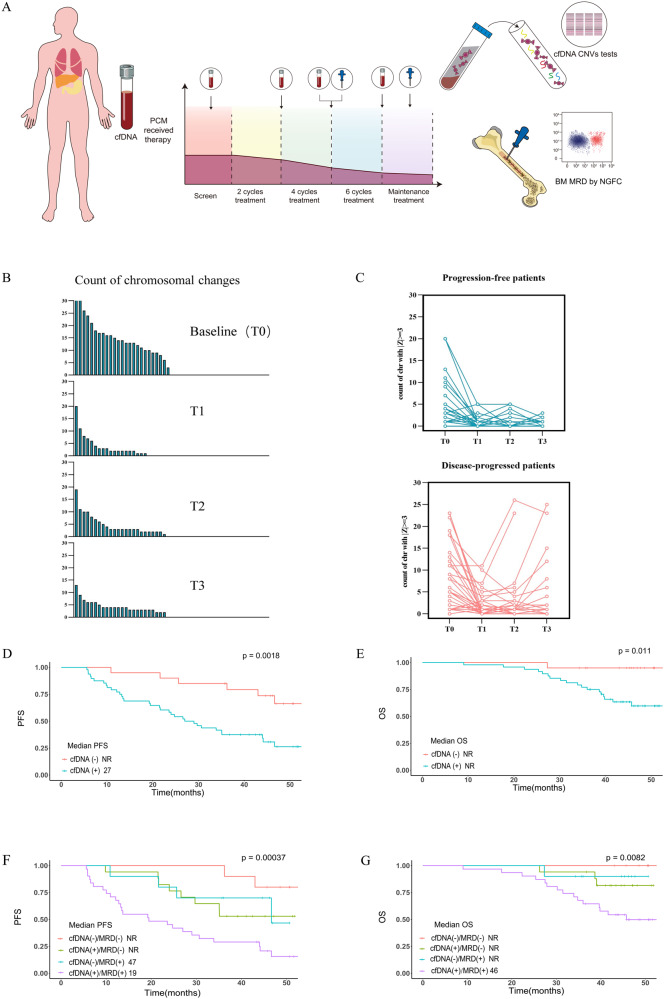
Time course of cfDNA aberrations. **A** Study flow diagram. Plasma samples were collected before, during and at the end of induction therapy. MRD was measured after 4 cycles of induction therapy, before an auto-transplant, or at the time of suspected complete remission every year thereafter. **B** Significant chromosomal aberrations (|*Z*| ≥ 3) detected before and during therapy. **C** cfDNA CNV changes (measured by maximum |*Z*| values) in progression free subjects and disease-progressed subjects. **D**, **E** Kaplan–Meier curves showing PFS (**D**) and OS (**E**) of cfDNA CNV-positive or -negative subjects. **F**, **G** Kaplan–Meier curves showing the PFS (**F**) and OS (**G**) of subjects based on combining cfDNA-CNV and interim NGFC MRD-test results. cfDNA cell-free DNA, CNVs copy number variations, MRD measurable residual disease, PCM plasma cell myeloma, BM bone marrow, NGFC next-generation flow cytometry, PFS progression-free survival, OS overall survival.

So-called *liquid biopsy* using cell-free DNA (cfDNA) is a non-invasive technique for diagnosis, evaluation, and/or monitoring of diverse cancers including hematological cancers [8, 9]. We developed an ultra-sensitive chromosome aneuploidy detector (UCAD) to profile genome-wide chromosomal instability through low-coverage whole-genome sequencing (LC-WGS) of cfDNA and used it to detect and monitor MRD in 242 longitudinal plasma cfDNA samples from 68 newly-diagnosed subjects with PCM treated at Shanghai Changzheng Hospital between March 2018 and December 2021 enrolled in a prospective trial (NCT04122092). We also compared the accuracy of this technique with MRD-testing by NGFC using the EURO-flow 8-color two-tube method. Diagnosis and response were assessed according to the International Myeloma Working Group (IMWG) criteria [10]. Subjects had blood and bone marrow sampling at diagnosis and progression to determine chromosomal aberrations. Induction therapy was bortezomib-based regimen, including lenalidomide, bortezomib and dexamethasone (VRD) or bortezomib, cyclophosphamide and dexamethasone (CBD). 29 subjects subsequently received an auto-transplant and posttransplant therapy with lenalidomide with or without bortezomib. We collected plasma samples at baseline (T_0_), at the end of induction cycles 2, 4, and 6 and at disease progression (Fig. 1A). Transplant recipients had additional samples taken immediately pretransplant. Total genomic DNA and cfDNA were isolated from plasma using the Circulating Nucleic Acid kit (Qiagen, Valencia, CA, USA). Segment copy number and tumor fraction (TFx) were derived using the customized UCAD workflow. Cell-free DNA from plasma samples was analyzed using the Illumina X 10 system. The detailed procedure of Low-coverage whole-genome sequencing and additional related methods details are provided in the Supplementary Method.

PFS and OS were estimated by using the Kaplan–Meier method. Data were analyzed with SPSS22.0 or R software version 3.4.3 (R Foundation for Statistical Computing, Auckland, New Zealand). *P*-values (two-tailed) less than 0.05 were set as the threshold for statistical significance.

Baseline clinical characteristics of subjects were showed in Table 1. Median age was 59 years (Interquartile Range [IQR], 54–65 years). 3 were Durie–Salmon (D-S) stage-II and 65, stage-III. 12 were revised International Staging System (R-ISS) stage-I, 52, stage-II and 4, stage-III. 18 had extra-medullary disease and 44, a high-risk cytogenetic profile defined as del(17p), 1q21gains, t(4;14) and/or t(14;16). Median follow-up is 47 months (IQR, 44–51 months).

**Table 1:** Subject co-variates (*N* – 68).

		N
Male		35
Age at diagnosis (years)	Median (range)	59 (34–74)
	<65	49
	≥65	19
D–S stage	I	0
	II	3
	III	65
ISS stage	I	18
	II	36
	III	14
R-ISS stage	I	12
	II	52
	III	4
Extra-medullary plasmacytoma		18
BM plasma cells (%)	≥50	17
Hemoglobin (g/L)	<100	27
Platelets (10E + 9/L)	<100	2
Serum LDH (U/L)	≥245	17
Serum creatinine (mg/dL)	≥2	7
Serum calcium (mmol/L)	≥2.65	12
ALB level (mg/dL)	<35	27
β_2_M (mmol/L)	≥3.5	37
M-protein	IgG	23
	IgM	0
	IgA	19
	IgD	5
	Light-chain	17
	Non-secretory	4
Light-chain	Kappa	38
	Lambda	26
	Non-secretory	4
sFLCR	0.01–100	33
	≤0.01, ≥100	35
del(13q)		17
del(17p)		3
1q21 gain		28
t(4;14)		13
t(11;14)		5
t(14;16)		0
Double hit		5
Triple hit		1

*D–S* Durie–Salmon Staging System, *ISS* International Staging System, *R-ISS* Revised International Staging System, *LDH* lactate dehydrogenase, *ALB* albumin, *β*_*2*_*M* beta-2 microglobulin, *sFLCR* serum-free light-chain ratio.

We did a genome-wide overview of copy number variants (CNVs) of by UCAD (Fig. S1). Chromosome breakpoints were frequently identified on centromeres resulting in chromosome arm imbalances. Averaged baseline data are displayed in Fig. S1A. In contrast, no abnormal CNVs were found in healthy controls (Fig. S1B). The most frequently identified CNVs in decreasing frequency were gains on chromosome 1q, 9, 15, 3, 7, 2, 5q, 8 and 21, including sites of *MYC* (8q) and *MCL1* (1q). Losses on chromosomes 13, 14, 12, 1p, 6, 16, 4, 5q, 20, 8, 10, 22, 17p, 7, and 11 were also identified including sites of *DLC1* (8p), *DKK2* (4q), *PTEN* (10q) and *TP53* (17p; Table S1).

59 subjects had ≥1 chromosome abnormality (|Z| ≥ 3) at baseline, 44 had ≥2 abnormalities and 9, no abnormality. Notably, the frequency of R-ISS stage-III or stage-II in subjects who had ≥1 chromosome abnormality at baseline, was significantly higher than those who had no CNVs (*P* = 0.04) (Table S2). There was no significant correlation between baseline CNVs and sex, age, D–S stage, serum free light-chain (sFLC) ratio, percentage bone marrow plasma cells, extra-medullary plasmacytoma or heavy- or light-chain type. Median PFS of subjects with ≥1 baseline CNV was 30 months (95% Confidence Interval [CI], 20, 41 months) compared with those who had no baseline CNVs, not reached but must exceed 34 months (HR = 10.03 [1.37, 73.50]; *P* = 0.005; Fig. S2A; Table S3). Corresponding 3-year OS rates were 100% compared with 78% (68, 89%; Fig. S2B; Table S3). In multi-variable analyses there was no significant correlation between baseline CNVs and PFS.

Next, we studied cfDNA samples during therapy. We found decreasing chromosome abnormalities were associated with therapy response **(**Fig. 1B, C; Fig. S1C). There were three categories of CNV changes from baseline in subjects with ≥1 baseline CNV after starting therapy. (1) a decrease in CNVs after two courses followed by further decreases (cohort 1; *N* = 27); (2) a decrease in CNVs after two courses followed by increases thereafter (cohort 2; *N* = 25); and (3) no change or an increase in CNVs after 2 courses followed by a subsequent increase in the former (cohort 3; *N* = 7). In 59 subjects who had ≥1 CNV at baseline, corresponding PFSs were 43 months (31, 55 months), 31 months (20, 41 months); and 7 months (6, 9 months; *P*-values = 0.001; Fig. S2C). Corresponding OSs were not reached but must exceed 45 months, not reached but must exceed 41 months and 27 months (22, 32 months; *P-*values = 0.04; Fig. S2D).

We subsequently determined the prognostic impact of monitoring chromosome profiles during therapy. 20 subjects had no CNVs in cfDNA samples after therapy regardless of baseline values (designated CNV-negative). The other 48 had ≥1 CNV (|Z| ≥ 3) in cfDNA samples during follow-up and were designated CNV-positive. Median PFS of subjects who became CNV-negative was not reached but must exceed 46 months compared with those who remained or became positive, 27 months (20, 35 months; *P* = 0.002; Fig. 1D). 3-year OS rates of these cohorts were 95% (86, 100%) and 75% (64, 88%; Fig. 1E). HR for PFS = 3.42 (1.41, 8.35; *P* = 0.006; Table S3; Table S4**)**. In multi-variable analyses a positive CNV-test correlated with an increased risk of death (HR = 7.21 [0.96, 54.28]; *P* = 0.06); Table S4).

27 subjects had a negative NGFC-test for MRD. 10 were cfDNA CNV-negative at simultaneous testing and 17, -positive (Table S5). NGFC-negative subjects had significantly longer PFS, not reached but must exceed 43 months, compared with 26 months (16, 35 months) in subjects NGFC-positive (HR = 2.87 [1.39, 5.90], *P* = 0.003; Table S3). In NGFC-negative subjects those cfDNA CNV-negative had a median PFS not reached but must exceed 43 months compared with those who were -positive, not reached but must exceed 35 months (*P* = 0.09). In NGFC-positive subjects those also CNV-positive had a median PFS of 19 months (7,32 months) compared with those who were -negative, not reached but must exceed 47 months (*P* = 0.023; Fig. 1F). Corresponding 3-year OS rates were 100% in subjects negative of both tests, 81% in subjects NGFC-negative but cfDNA CNV-positive and 65% in subjects positive in both tests (Fig. 1G). CNV-positive subjects with a NGFC-negative test had a HR of 3.63 (0.75, 17.5; *P* = 0.09) compared with those who were negative in both tests. Amongst subjects who were NGFC-test negative there were no significant differences in baseline covariates between those cfDNA CNV-positive and -negative (Table S6).

Liquid biopsy techniques in PCM include quantifying blood plasma cells, concentration of cfDNA or circulating myeloma DNA (ctDNA) and analyzing mutation topography by next-generation sequencing [11–13]. There are few data on the quantitation of CNVs in cfDNA samples [14, 15]. We used an UCAD to identify CNVs in cfDNA plasma samples, and obtained longitudinal collections which allowed us to monitor CNV changes of PCM in response to treatment. We found dynamic cfDNA CNVs detection was a better predictor of prognosis than baseline cfDNA CNVs, and dynamics cfDNA-CNV analysis during therapy was more sensitive than interim MRD responses by NGFC. Our study has important limitations. 1st, we had few subjects requiring validation of our findings. 2nd, CNVs based on LC-WGS do not provide potentially important cytogenetic data such as t(4;14), t(11;14) and t(14;16).

In summary, our data indicate changes from baseline levels of cfDNA-CNVs after therapy increases prediction accuracy of PFS and OS in subjects with newly-diagnosed PCM and is more accurate than MRD-testing by NGFC. Combining plasma cfDNA-CNV analysis with standard approaches for MRD detection may usefully contribute to the prognostic analysis of PCM. If validated, this approach may help physicians with therapy decision-making.

### Supplementary information

supplemental information-clean version
