# Supplementary material for: Cell-free DNA chromosome copy number variations predict outcomes in plasma cell myeloma
Source: Blood Cancer J. 2023 Sep 5;13(1):136. doi: 10.1038/s41408-023-00904-9 (PMC10480144; doi:10.1038/s41408-023-00904-9)
Supplement: Supplementary file 1 — supplemental information-clean version [file 41408_2023_904_MOESM1_ESM.docx]

**Supplemental Information**

**Supplementary Materials and Methods 1**

**Supplementary Figures and Figure Legends7**

**Supplementary Tables 9**

**Supplementary Materials and Methods**

**Patients and ethics statement**

The cohort consisted of 68 newly-diagnosed subjects with PCM treated at Shanghai Changzheng hospital between March 2018 and December 2021, partly enrolled in NCT04122092. The study was conducted in accordance with the International Conference on Harmonization Guideline for Good Clinical Practice and the Declaration of Helsinki, and the study protocol was approved by the medical Ethics Committee. Written informed consent was obtained from each subject.

**DNA extraction**

Plasma samples were isolated from whole blood EDTA tubes by two-step centrifugation: 300 × g for 10 min and 3000 × g for 10 min. DNA was extracted from 2–6 mL plasma using the Circulating Nucleic Acid kit (Qiagen, Valencia, CA, USA) according to the manufacturer’s instructions

**Low-coverage whole-genome sequencing**

For low-coverage-whole genome sequencing (LC-WGS) libraries were prepared using the Kapa Hyper Prep kit with custom adapters (IDT and Broad Institute). A median of 5 ng of input cfDNA (range, 3–20 ng) or approximately 1,000 to 7,000 haploid genome equivalents, was studied. Up to 22 libraries were pooled and sequenced using 150 bp paired-end runs over 1 × lane on the HiSeq X 10 system (Illumina, San Diego, CA，USA). Segment copy number and tumor fraction (TFx) were derived using the customized UCAD workflow. Samples were excluded if the median absolute deviation (MAD) of copy ratios (2log_2_ ratio) between adjacent bins genome-wide was 0.20 indicating poor-quality sequence data.

**Fluorescence *in situ* hybridization (FISH)**

FISH was done using standardized protocols with commercially available probes (Abbott Molecular, Inc., Chicago, Illinois, USA). Two hundred nuclei were analyzed using a fluorescent light microscope (Olympus Corporation, Tokyo, Japan). Cut-off levels for positive values for each probe were: 10% for fusion and 20% for numerical abnormalities according to European Myeloma Network FISH workshops recommendations. Subjects with t(4;14), t(14;16), 1q21 gain, and/or del(17p13) were classified as high-risk and others as standard-risk, according to Mayo Stratification of Myeloma and Risk-Adapted Therapy (mSMART)3.0.

**Next generation multi-parameter flow cytometry**

We evaluated MRD in bone marrow samples as described using the NGFC sample preparation protocol (2-tube-8-color panel for a total of 12 different markers) developed by EuroFlow platform (MMMRD panel composition) using a Beckman Coulter Navios flow cytometer and Cytognos Infnicyt™ software (Beckman Coulter, Inc. Brea CA USA). Intra-cytoplasmic light-chain expression was determined using kappa and lambda antibodies (Dako, Glostrup, Denmark). Abnormal light-chain ratios were defined as < 0.5 and > 4. Acquisition of ≥ 10E+7 cells/sample was required to achieve a minimum sensitivity of 10E-6. MRD was studied at the end of 4 courses of induction therapy, before an auto-transplant and/or at the time of clinical complete remission and every 6-12months during follow-up. Subjects were scored as MRD-test-positive if the MRD assessment was positive, indeterminate, or not done.

**Data visualization**

Cell-free DNA from plasma samples were analyzed using the Illumina X 10 system. At least 10 M paired reads were collected for each sample. Reads were mapped to human reference genome hg19 and genomic coverage analyzed using SAMtools mpileup software. Average coverage was calculated for each 200k bin. Z-scores were normalized for each bin using the formula below: ${coverage}_{normalized}=\frac{{coverege}_{raw}-mean({coverage}_{controls, raw})}{stdev\left( {coverage}_{controls, raw} \right)}$ (Formula 1)

where ${coverege}_{raw}$is the raw coverage obtained from sequencing and${coverage}_{controls, raw}$is the raw coverage from the technique control samples with matched laboratory protocols.

More specifically, Z-scores for chr1p, 1q, 2, 3, 4, 5p, 5q, 6, 7, 8, 9, 10, 11, 12, 13, 14, 15, 16, 17p, 17, 18, 19, 20, 21, and 22 were calculated by normalizing to healthy controls, using the following formula:

$Z=\frac{V_{tumor}-average\left( V_{control} \right)}{stdev\left( V_{control} \right)}$ (Formula 2)

where $V_{tumor}$ is the normalized sequencing coverage of tumor sample and $V_{control}$ is the normalized sequencing coverage of control sample in the same genomic region. A copy number gain was defined as Z ≥ 3, and loss as Z ≤ -3. |Z| measure how many standard deviations a data point is above or below the mean.

The circular binary segmentation algorithm from the R package DNACopy was used to identify significant genomic breakpoints and genomic segments showing copy number changes.

**Statistics**

Descriptive statistics included mean ± standard deviation (SD) or medians with ranges for continuous variables and counts with percentages for categorical variables. Continuous variables were analyzed with the Kruskal-Wallis or Wilcoxon rank sum tests. Categorical variables were compared by using Chi-square test and Hazard Ratios (HRs) or Odds Ratios (ORs) with 95% Confidence Intervals (CIs). Progression-free survival (PFS) was defined as the interval from therapy start to progression, death, withdrawal of consent or loss to follow-up. Overall survival (OS) was defined as the interval from therapy start to death, withdrawal of consent or loss to follow-up. PFS and OS were estimated by using the Kaplan–Meier method. Data were analyzed with SPSS22.0 or R software version 3.4.3 (R Foundation for Statistical Computing, Auckland, New Zealand). *P*-values (two-tailed) less than 0.05 were set as the threshold for statistical significance.

**Supplementary Figures and Figure Legends**

**Supplementary Figure 1. Genomic profiling of plasma cell-free DNA from subjects with PCM.** Multiple chromosomal aberrations were found in cfDNA from 68 newly-diagnosed subjects with PCM (A) but not in cfDNA from non-tumor controls (B). Baseline chromosome aberrations were more frequent in disease-progressed patients during follow-up (C).

Abbreviations: cfDNA, cell-free DNA; PCM: plasma cell myeloma.

**
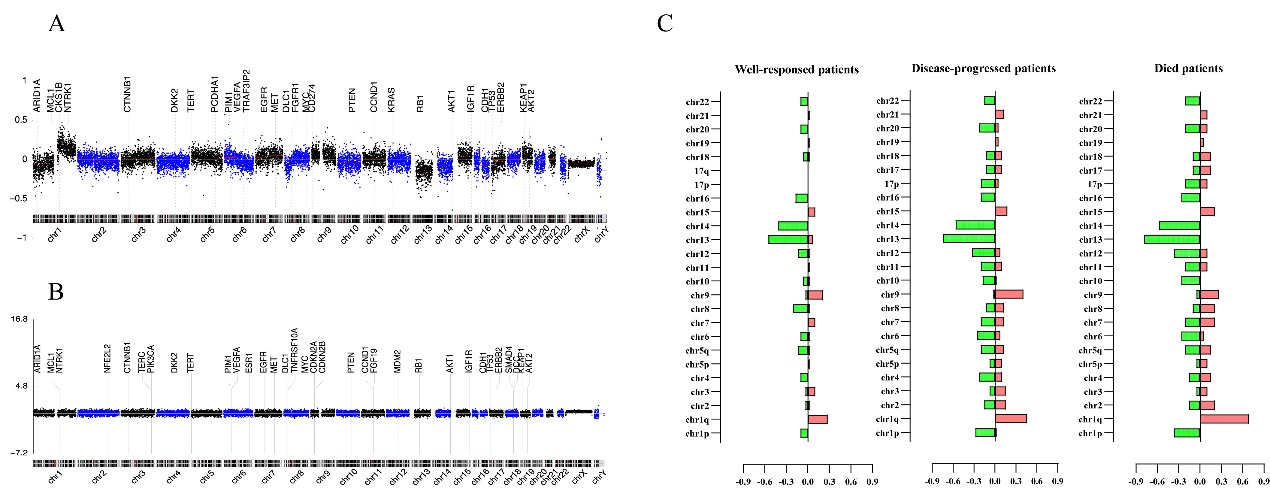
**

**Supplementary Figure 2. Progression free survival and overall survival.**

(A, B) Kaplan-Meier curves showing PFS (A) and OS (B) in subjects cfDNA CNV-positive (≥ 1 chromosome abnormality) or -negative at baseline (T_0_). (C, D) Kaplan-Meier curves showing PFS (C) and OS (D) in subjects with different CNV changes from baseline after starting therapy: cohort 1(a continual decrease in CNVs over 2 courses), cohort 2(initial decrease then increases CNVs after therapy) and cohort 3(no change or an increase in CNVs after therapy).

Abbreviations: PFS, progression-free survival; OS, overall survival; cfDNA, cell-free DNA; CNVs, copy number variations.


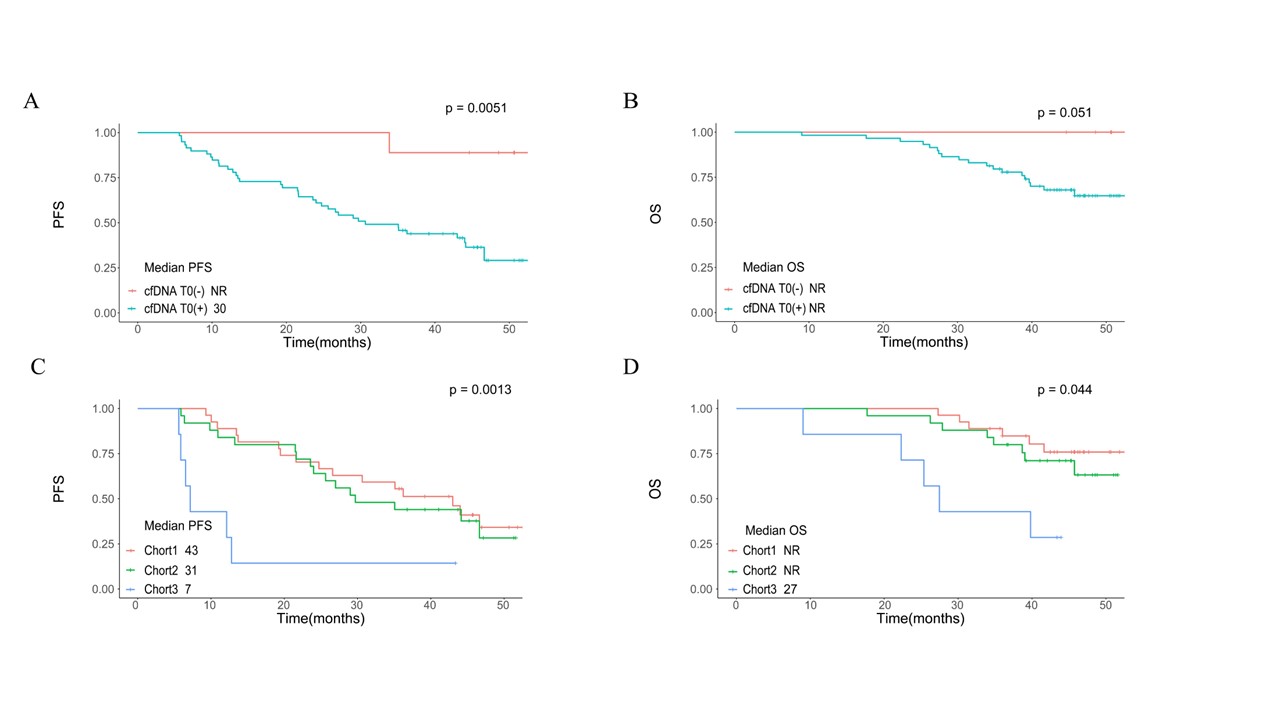


**Supplementary Tables**

**Supplementary Table1. Summary of copy number changes in 68 subjects with PCM samples at baseline.**

| Chromosome | Deletions (%) | Chromosome | Gains (%) |
| --- | --- | --- | --- |
| chr13 | 66.18% | chr1q | 38.24% |
| chr14 | 50.00% | chr9 | 32.35% |
| chr12 | 25.00% | chr15 | 14.71% |
| chr1p | 20.59% | chr3 | 13.24% |
| chr6 | 19.12% | chr7 | 11.76% |
| chr16 | 19.12% | chr2 | 10.29% |
| chr4 | 17.65% | chr5q | 8.82% |
| chr5q | 17.65% | chr8 | 8.82% |
| chr20 | 17.65% | chr21 | 8.82% |
| chr8 | 16.18% | chr11 | 7.35% |
| chr10 | 13.24% | chr18 | 7.35% |
| chr22 | 13.24% | chr5p | 7.35% |
| 17p | 11.76% | chr12 | 5.88% |
| chr7 | 11.76% | chr6 | 5.88% |
| chr11 | 11.76% | chr4 | 5.88% |
| chr2 | 10.29% | 17q | 5.88% |
| chr18 | 10.29% | chr19 | 4.41% |
| 17q | 7.35% | chr13 | 2.94% |
| chr3 | 5.88% | chr20 | 2.94% |
| chr5p | 4.41% | chr10 | 2.94% |
| chr9 | 2.94% | 17p | 2.94% |
|  |  | chr1p | 1.47% |

**Supplementary Table2. Subject characteristics separated by baseline cfDNA status.**

| Variable |  | cfDNA CNV(T0)^+^ (N = 59, %) | cfDNA CNV(T0)^-^ (N = 9, %) | *P*-value |
| --- | --- | --- | --- | --- |
| Sex | Male | 28(47.4) | 7(77.8) | 0.089 |
|  | Female | 31(52.6) | 2(22.2) |  |
| Age at diagnosis, years | < 65 | 41(69.5) | 8(88.9) | 0.216 |
|  | ≥ 65 | 18(30.5) | 1(11.1) |  |
| DS stage | I | 0(0) | 0(0) | 0.351 |
|  | II | 2(3.4) | 1(11.1) |  |
|  | III | 57(96.6) | 8(88.9) |  |
| ISS stage | I | 14(23.7) | 4(44.4) | 0.362 |
|  | II | 33(56.0) | 3(33.3) |  |
|  | III | 12(20.3) | 2(22.2) |  |
| R-ISS stage | I | 8(13.6) | 4(44.4) | 0.047 |
|  | II | 48(81.3) | 4(44.4) |  |
|  | III | 3(5.1) | 1(11.1) |  |
| Extramedullary plasmacytoma | No | 41(69.5) | 9(100) | 0.051 |
|  | Yes | 18(30.5) | 0(0) |  |
| BM plasma cells (%) | < 50 | 44(74.6) | 7(77.8) | 0.601 |
|  | ≥ 50 | 15(25.4) | 2(22.2) |  |
| Hemoglobin level (g/L) | < 100 | 23(39.0) | 4(44.4) | 0.514 |
|  | ≥ 100 | 36(61.0) | 5(55.6) |  |
| Platelet count (10E+9/L) | < 100 | 2(3.4) | 0(0) | 0.751 |
|  | ≥ 100 | 57(96.6) | 9(100) |  |
| Serum LDH (U/L) | < 245 | 42(71.2) | 9(100) | 0.062 |
|  | ≥ 245 | 17(28.8) | 0(0) |  |
| Serum creatinine level (mg/dL) | < 2 | 53(89.8) | 8(88.9) | 0.648 |
|  | ≥ 2 | 6(10.2) | 1(11.1) |  |
| Serum calcium level (mmol/L) | < 2.65 | 47(79.7) | 9(100) | 0.154 |
|  | ≥ 2.65 | 12(20.3) | 0(0) |  |
| ALB level (mg/dL) | < 35 | 24(40.7) | 3(33.3) | 0.486 |
|  | ≥ 35 | 35(59.3) | 6(66.7) |  |
| β2M level (mmol/L) | < 3.5 | 26(44.1) | 5(55.6) | 0.386 |
|  | ≥ 3.5 | 33(55.9) | 4(44.4) |  |
| M-protein restriction | IgG | 19(32.2) | 4(44.4) | 0.831 |
|  | IgM | 0(0) | 0(0) |  |
|  | IgA | 17(28.8) | 2(22.2) |  |
|  | IgD | 5(8.5) | 0(0) |  |
|  | Light chain | 14(23.7) | 3(33.3) |  |
|  | Non-secretory | 4(6.8) | 0(0) |  |
| Light chain restriction | Kappa | 35(59.3) | 3(33.3) | 0.154 |
|  | Lambda | 20(33.9) | 6(66.7) |  |
|  | Non-secretory | 4(6.8) | 0(0) |  |
| sFLCR | 0.01–100 | 28(47.5) | 5(55.6) | 0.461 |
|  | ≤0.01, ≥ 100 | 31(52.5) | 4(44.4) |  |
| Del(13q) in FISH | No | 44(74.6) | 7(77.8) | 0.601 |
|  | Yes | 15(25.4) | 2(22.2) |  |
| Del(17p) in FISH | No | 56(94.9) | 9(100) | 0.649 |
|  | Yes | 3(5.1) | 0(0) |  |
| 1q21 gains in FISH | No | 35(59.3) | 5(55.6) | 0.553 |
|  | Yes | 24(40.7) | 4 (44.4) |  |
| t(4;14) in FISH | No | 48(81.4) | 7(77.8) | 0.550 |
|  | Yes | 11(18.6) | 2(22.2) |  |
| t(11;14) in FISH | No | 55(93.2) | 8(88.9) | 0.520 |
|  | Yes | 4(6.8) | 1(11.1) |  |
| t(14;16) in FISH | No | 59(100) | 9(100) | / |
|  | Yes | 0(0) | 0(0) |  |
| Double hit | No | 55(93.2) | 8(88.9) | 0.520 |
|  | Yes | 4(6.8) | 1(11.1) |  |
| Triple hit | No | 58(98.3) | 9(100) | 0.868 |
|  | Yes | 1(1.7) | 0(0) |  |

Abbreviations: DS, Durie-Salmon Staging System; ISS, International Staging System; R-ISS, Revised International Staging System; LDH, lactate dehydrogenase; ALB, albumin; β2M, beta2 microglobulin; sFLCR, serum-free light chain ratio.

**Supplementary Table 3. Single-parameter analyses of PFS and OS**

| Variables | PFS | | OS | |
| --- | --- | --- | --- | --- |
|  | Univariate OR (95% CI) | *P*-value | Univariate OR (95% CI) | *P*-value |
| Sex (female ref.) | 1.165 (0.617–2.199) | 0.638 | 1.506 (0.592–3.827) | 0.387 |
| Age (≤ 65 years ref.) | 0.617 (0.318–1.413) | 0.290 | 0.831 (0.299–2.308) | 0.722 |
| Serum creatinine level (< 2mg/dL ref.) | 1.183 (0.418–3.346) | 0.782 | 2.308(0.307–17.350) | 0.403 |
| ISS, III vs. II vs. I | 1.106 (0.715–1.712) | 0.651 | 1.236 (0.650–2.348) | 0.517 |
| R-ISS, III vs. II vs. I | 1.391(0.704–2.746) | 0.343 | 2.040 (0.745–5.590) | 0.171 |
| FISH high risk stratification (No ref.) | 0.938(0.500–1.761) | 0.843 | 1.033 (0.419–2.544) | 0.944 |
| del(17p) (No ref) | 5.230 (1.563–17.500) | 0.003 | 3.751 (0.894–16.285) | 0.058 |
| 1q21+ (No ref.) | 0.812 (0.426–1.548) | 0.525 | 1.131 (0.454–2.816) | 0.791 |
| Extra-medullary plasmacytoma (No ref.) | 2.700 (1.370–5.320) | 0.003 | 2.594 (1.038–6.486) | 0.034 |
| Baseline cfDNA-CNV-positive (Negative ref.) | 10.032 (1.369–73.499) | 0.005 | / | 0.051 |
| Follow-up cfDNA-CNV-positive (Negative ref.) | 3.691 (1.535–8.875) | 0.002 | 8.738 (1.165–65.519) | 0.011 |
| BM-MRD-positive (Negative ref.) | 2.867 (1.393–5.900) | 0.003 | 4.243 (1.235–14.579) | 0.012 |

Abbreviations: PFS, progression-free survival; OS, overall survival; ISS, International Staging System; R-ISS, Revised International Staging System; cfDNA, cell-free DNA; CNV, copy number variation; BM, bone marrow; MRD, measurable residual disease; ref, reference, as control group.

**Supplementary Table 4. Multi-variable analyses of PFS and OS**

| Variable |  | PFS | | OS | |
| --- | --- | --- | --- | --- | --- |
|  | n/N (%) | OR (95% CI) | *P*-value | OR (95% CI) | *P*-value |
| del(17p) (No ref) | 3/68(4.4%) | 4.167 (1.167–14.883) | 0.028 |  | ＞0.05 |
| 1q21+ (No ref.) | 28/68(41.2%) |  | ＞0.05 |  | ＞0.05 |
| Extra-medullary plasmacytoma (No ref.) | 18/68(26.5%) | 2.854 (1.417–5.748) | 0.003 |  | ＞0.05 |
| Baseline cfDNA-CNV-positive (Negative ref.) | 59/68(86.8%) |  | ＞0.05 |  | ＞0.05 |
| Follow-up cfDNA-CNV-positive (Negative ref.) | 48/68(70.6%) | 3.415 (1.413–8.353) | 0.006 | 7.208 (0.957–54.281) | 0.055 |
| BM-MRD-positive (Negative ref.) | 41/68(60.3%) | 2.141(1.022–4.488) | 0.044 | 3.506 (1.017–12.085) | 0.047 |

Abbreviations: PFS, progression-free survival; OS, overall survival; cfDNA, cell-free DNA; CNV, copy number variation; BM, bone marrow; MRD, measurable residual disease; ref, reference, as control group.

**Supplementary Table 5. Comparison between BM-MRD by NGFC and plasma cfDNA-CNV.**

|  |  | Plasma cfDNA | | *P-*value |
| --- | --- | --- | --- | --- |
|  |  | CNV-neg | CNV-pos |  |
| NGFC-BM | MRD-neg | 10 | 17 | 0.198 |
|  | MRD-pos | 10 | 31 |  |

Abbreviations: cfDNA, cell-free DNA; CNV, copy number variation; BM, bone marrow; MRD, measurable residual disease; NGFC, next-generation flow cytometry; neg, negative; pos, positive.

**Supplementary Table 6. MRD-negative by NGFC subject characteristics separated by cfDNA status.**

| Variable |  | cfDNA^-^MRD^-^ | cfDNA^+^MRD^-^ | *P*-value |
| --- | --- | --- | --- | --- |
| Sex | Male | 7(70) | 8(47.1) | 0.226 |
|  | Female | 3(30) | 9(52.9) |  |
| Age at diagnosis, years | < 65 | 8(80) | 11(64.7) | 0.349 |
|  | ≥ 65 | 2(20) | 6(35.3) |  |
| DS stage | I | 0(0) | 0(0) | 0.630 |
|  | II | 0(0) | 1(5.9) |  |
|  | III | 10(100) | 16(94.1) |  |
| ISS stage | I | 4(40) | 4(23.5) | 0.205 |
|  | II | 3(30) | 11(64.7) |  |
|  | III | 3(30) | 2(11.8) |  |
| R-ISS stage | I | 4(40) | 3(17.6) | 0.146 |
|  | II | 5(50) | 14(82.4) |  |
|  | III | 1(10) | 0(0) |  |
| Extramedullary plasmacytoma | No | 9(90) | 13(76.5) | 0.371 |
|  | Yes | 1(10) | 4(23.5) |  |
| Plasma cells of BM (%) | < 50 | 7(70) | 14(82.4) | 0.387 |
|  | ≥ 50 | 3(30) | 3(17.6) |  |
| Hemoglobin level (g/L) | < 100 | 3(30) | 14(82.4) | 0.387 |
|  | ≥ 100 | 7(70) | 3(17.6) |  |
| Platelet count (10E+9/L) | < 100 | 0(0) | 0(0) | / |
|  | ≥ 100 | 10(100) | 17(100) |  |
| Serum LDH (U/L) | < 245 | 9(90) | 13(76.5) | 0.371 |
|  | ≥ 245 | 1(10) | 4(23.5) |  |
| Serum creatinine level (mg/dL) | < 2 | 8(80) | 16(94.1) | 0.303 |
|  | ≥ 2 | 2(20) | 1(5.9) |  |
| Serum calcium level (mmol/L) | < 2.65 | 9(90) | 16(94.1) | 0.494 |
|  | ≥ 2.65 | 1(10) | 1(5.9) |  |
| ALB level (mg/dL) | < 35 | 7(70) | 13(76.5) | 0.525 |
|  | ≥ 35 | 3(30) | 4(23.5) |  |
| β2M level (mmol/L) | < 3.5 | 7(70) | 10(58.8) | 0.437 |
|  | ≥ 3.5 | 3(30) | 7(41.2) |  |
| M-protein restriction | IgG | 3(30) | 5(29.4) | 0.575 |
|  | IgM | 0(0) | 0(0) |  |
|  | IgA | 3(30) | 2(11.8) |  |
|  | IgD | 0(0) | 1(5.9) |  |
|  | Light chain | 4(40) | 7(41.2) |  |
|  | Non-secretory | 0(0) | 2(11.8) |  |
| Light chain restriction | Kappa | 5(50) | 9(52.9) | 0.467 |
|  | Lambda | 5(50) | 6(35.3) |  |
|  | Non-secretory | 0(0) | 2(11.8) |  |
| sFLCR | 0.01–100 | 4(40) | 9(52.9) | 0.402 |
|  | ≤ 0.01, ≥ 100 | 6(60) | 8(47.1) |  |
| Del(13q) in FISH | No | 8(80) | 12(70.6) | 0.512 |
|  | Yes | 2(20) | 5(29.4) |  |
| Del(17p) in FISH | No | 10(100) | 17(100) | / |
|  | Yes | 0(0) | 0(0) |  |
| 1q21 gains in FISH | No | 8(80) | 11(64.7) | 0.349 |
|  | Yes | 2(20) | 6(35.3) |  |
| t(4;14) in FISH | No | 8(80) | 14(82.4) | 0.629 |
|  | Yes | 2(20) | 3(17.6) |  |
| t(11;14) in FISH | No | 9(90) | 16(94.1) | 0.613 |
|  | Yes | 1(10) | 1(5.9) |  |
| t(14;16) in FISH | No | 10(100) | 17(100) | / |
|  | Yes | 0(0) | 0(0) |  |
| Double hit | No | 9(90) | 17(100) | 0.370 |
|  | Yes | 1(10) | 0(0) |  |
| Triple hit | No | 10(100) | 17(100) | / |
|  | Yes | 0(0) | 0(0) |  |

Abbreviations: MRD, measurable residual disease; NGPC, next-generation flow cytometry; DS, Durie-Salmon Staging System; ISS, International Staging System; R-ISS, Revised International Staging System; LDH, lactate dehydrogenase; ALB, albumin; β2M, beta2 microglobulin; sFLCR, serum-free light chain ratio.
